# Fracture Resistance of Commercial and Novel Ceramic-Reinforced Polymer Crowns with Luting Cements of Varying Elastic Modulus

**DOI:** 10.3390/polym18010025

**Published:** 2025-12-22

**Authors:** Naluemol Sriprasert, Nantawan Krajangta, Thanakorn Wasanapiarnpong, Pavinee Padipatvuthikul Didron, Thanasak Rakmanee

**Affiliations:** 1 Department of Restorative and Esthetic Dentistry, Faculty of Dentistry, Thammasat University, Patum Thani 12120, Thailand; naluemol.s@rsu.ac.th (N.S.); knantawa@tu.ac.th (N.K.); 2 College of Dental Medicine, Rangsit University, Patum Thani 12120, Thailand; 3 Upcycled Materials from Industrial and Agricultural Wastes Research Unit, Department of Materials Science, Faculty of Science, Chulalongkorn University, Bangkok 10110, Thailand; thanakorn.w@chula.ac.th; 4 Department of General Dentistry, Faculty of Dentistry, Srinakharinwirot University, Bangkok 10110, Thailand; pavinee.didron@gmail.com

**Keywords:** ceramic-reinforced polymer, resin nanoceramic, luting cement, fracture resistance, elastic modulus

## Abstract

This in vitro study investigated the fracture resistance of three ceramic-reinforced polymer (CRP) crowns—Cerasmart^®^ 270 (CE; milled), VarseoSmile Crown Plus^®^ (VS; 3D-printed) and the newly developed Hassawat-01 (HS; 3D-printed)—luted with cements of different elastic moduli. The principal hypothesis was that neither the CRP type nor the modulus of cement would significantly affect fracture resistance. Ninety-nine mandibular first molar resin dies were restored with 1 mm thick CE, VS, or HS crowns (n = 33 each) and luted with Maxcem Elite^®^, RelyX Unicem^®^, or Ketac Cem^®^ (n = 11 per subgroup). Occlusal cement morphology was evaluated using Micro-CT. Fracture resistance was measured using a universal testing machine. Crowns luted with Maxcem or RelyX withstood forces >2000 N without visible failure. Ketac-luted crowns showed reduced fracture resistance. CE-Ketac fractured in 4 of 11 specimens. VS-Ketac exhibited cracks or complete fractures (1795.2 ± 156.7 N), whereas HS-Ketac showed only superficial cracking (1732.6 ± 127.3 N). CRP crowns luted with lower-modulus resin cements demonstrated superior fracture resistance compared with those luted with glass-ionomer. VS exhibited both cracking and occasional complete fractures, whereas HS exhibited only surface cracking. All materials withstood loads greater than typical masticatory forces, supporting HS as a promising alternative within the CRP.

## 1. Introduction

Ceramic-reinforced polymers (CRPs) have become an important class of hybrid restorative materials, combining the toughness, reparability, and photopolymerization advantages of cross-linked polymers with the strength, stiffness, and esthetics provided by inorganic ceramic fillers [1,2,3,4,5,6]. CRPs are typically classified into polymer-infiltrated ceramic networks (PICNs) and resin nanoceramics (RNCs). PICNs contain a porous silanized ceramic scaffold—usually leucite- or zirconia-based—into which a polymer infiltrates to form an interpenetrating network, whereas RNCs incorporate nanoscale silica or barium-glass (BG) fillers within a highly cross-linked resin matrix [4,5,7]. Among commercial RNCs, Cerasmart^®^ 270 (CE), which contains 71 wt.% ceramic fillers (20 nm silica and 300 nm barium glass), has shown favorable mechanical behavior and clinical performance [5].

Although CAD/CAM milling of pre-polymerized CRP blocks provides consistent material quality, it is inherently wasteful and can introduce microcracks during machining. Additive manufacturing, particularly digital light processing (DLP), has therefore gained attention for its efficiency, greater control over polymerization, and potential for improved filler distribution [8,9,10,11,12,13]. Among DLP-printable CRP materials, VarseoSmile Crown Plus^®^ (VS) incorporates 30–50 wt.% silanized ceramic fillers and is approved for single-unit restorations. However, its relatively low filler content raises concerns about long-term stability and strength [14,15,16]. To address these limitations, a novel DLP-printable CRP, Hassawat-01 (HS), was developed using alumina as the reinforcing phase. Alumina was selected because of its high hardness, wear and corrosion resistance, color stability, and established biocompatibility in polymer-ceramic medical composites [13,17,18]. Urethane acrylate (UA) was chosen as the organic matrix because of its favorable cross-link density, toughness, photoreactivity, and reduced polymerization shrinkage, characteristics that support DLP printing and load-bearing applications [19,20,21]. Previous studies indicate that incorporating nano- to microscale alumina into photocurable polymers can enhance flexural strength, modulus, hardness, and optical stability [8,9,13]. Based on these structure–property relationships, HS was formulated with 38.5 wt.% 0.7 µm alumina fillers and 61.5 wt.% UA-based resin to achieve a balance between mechanical reinforcement and printability.

Despite early concerns about their long-term mechanical reliability, several in vitro studies have shown that CRP materials can withstand clinically relevant occlusal forces [22,23,24,25]. Donmez and Okutan reported acceptable performance of CRP crowns, with mean fracture loads of 1274 ± 135 N [26]. Suksuphan et al. showed that VS crowns cemented with RelyX Unicem^®^ had fracture resistance values of 1480 to 1747 N, whereas CE crowns exceeded 2000 N [27]. Zimmermann et al. reported fracture loads spanning 571 to 1580 N for CAD/CAM hybrid ceramics and RNCs, including CE [28]. Other studies reported similar findings, with CE crowns achieving fracture loads of 1000.9 to 1111.3 N, depending on occlusal thickness [23]. These values exceed typical adult bite forces of 315 to 806 N (men: 453 to 806 N; women: 315 to 567 N) [24,25], suggesting that CRPs, when properly luted, may be suitable for posterior restorations.

However, the mechanical performance of CRP crowns is not determined by material composition alone. The elastic modulus of the luting cement strongly influences stress distribution across the crown, cement, substrate complex, affecting both stress concentration and failure patterns [29,30]. Finite element analyses identify cement modulus as a critical factor governing intra-crown stresses, and they generally recommend intermediate-modulus or modulus-compatible cements for high-modulus ceramics [31,32,33]. These findings align with the cement-adaptation theory, which states that modulus-compatible cements transmit occlusal loads more effectively to the tooth and minimize residual stress within the crown [34]. Pairing IPS e.max CAD^®^ with a higher-modulus resin cement (NX3^®^) reduced peak stress in cervical enamel, even though internal cement stress increased, which lowered the likelihood of adhesive failure [35]. Taken together, these findings support the use of intermediate-to-high modulus resin cements, with good adaptation and controlled film thickness, to optimize fracture resistance in ceramic restorations [29,30,31,32,33,34,35,36,37,38,39,40]. However, these recommendations may not translate directly to low-modulus CRPs, whose elasticity can be five to ten times lower than that of lithium disilicate or zirconia, and for which experimental data remain limited.

Accordingly, the present study investigated the influence of luting cement modulus on the fracture resistance of crowns fabricated from three CRP systems. Those include CE, a milled and highly filled RNC; VS, a moderately filled DLP-printable CRP; and HS, a novel alumina-reinforced DLP-printable CRP with a UA matrix. Three cements with low to high elastic moduli, Maxcem Elite^®^ (approx. 4 GPa), RelyX Unicem^®^ (approx. 13 GPa), and Ketac Cem^®^ (approx. 20 GPa), were used to evaluate how modulus compatibility affects load transfer and failure behavior. Micro-CT was also used as a qualitative tool to visualize internal cement morphology and support interpretation of interfacial integrity. Beyond the primary objective, the study also compared the mechanical performance of the newly developed HS material with established commercial CRPs. The null hypothesis stated that neither the type of CRP material, including HS relative to CE and VS, nor the modulus of the luting cement would significantly affect fracture resistance.

## 2. Materials and Methods

### 2.1. Study Design

Ninety-nine mandibular first molar CRP crowns were fabricated using CAD/CAM technology. Three materials were tested: a milled resin nanoceramic (Cerasmart^®^ 270; CE) and two DLP-printed CRPs (VarseoSmile Crown Plus^®^; VS and the experimental alumina-reinforced Hassawat-01; HS) with 33 specimens per group. CE crowns were milled using a CARES M Series unit (Straumann, Basel, Switzerland). VS and HS crowns were fabricated with a DLP printer (Freeform Pro 2; ASIGA, Sydney, NSW, Australia) from CAD-generated STL files. Printed crowns were cleaned in 96% ethanol using an ultrasonic bath, air-dried, and post-cured according to manufacturer instructions [41]. Internal surfaces were air-abraded with 50 μm Al_2_O_3_ at 1.5 bar from a distance of 10 mm for 10 s, air-dried, and treated with a silane coupling agent (RelyX Ceramic Primer^®^; 3M ESPE, St. Paul, MN, USA) [14,42]. Each group was divided into three cementation subgroups (n = 11) using luting agents of differing elastic moduli: Maxcem Elite^®^ (approx. 4 GPa), RelyX Unicem^®^ (approx. 13 GPa), and Ketac Cem^®^ (approx. 20 GPa), resulting in nine experimental conditions. Sample size was calculated using G*Power v3.1 (HHU Düsseldorf) with α = 0.05 and power = 0.80. An effect size derived from a previous study [27] confirmed that 11 specimens per subgroup were adequate to detect significant differences.

**Figure 1:**
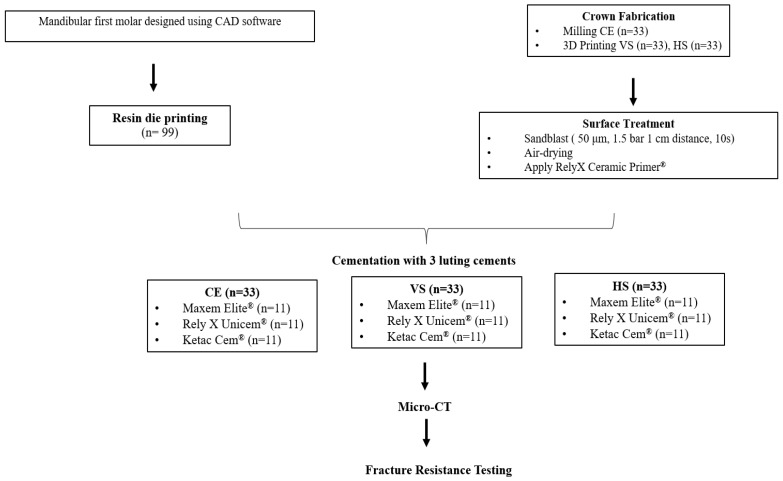
Conceptual framework of the study design.

Crowns were bonded to standardized 3D-printed resin dies (Rigid 10K; Formlabs Inc., Somerville, MA, USA) and stored at 37 °C for 24 h to allow complete cement polymerization. Cement interfaces were evaluated by micro-computed tomography (SkyScan 1172; Bruker microCT, Kontich, Belgium), and fracture resistance was tested using a universal testing machine (Shimadzu Corporation, Kyoto, Japan) under axial compression at 1 mm/min until failure [27]. The testing machine had a maximum force limit of 2000 N, so only specimens with measurable failure loads at or below this limit were included in the statistical analysis. Specimens exceeding 2000 N were classified as right-censored and excluded. Material compositions and the conceptual framework are summarized in Table 1 and Figure 1, respectively.

### 2.2. Virtual Design of Resin Die and CRP Crown Preparation

**Figure 2:**
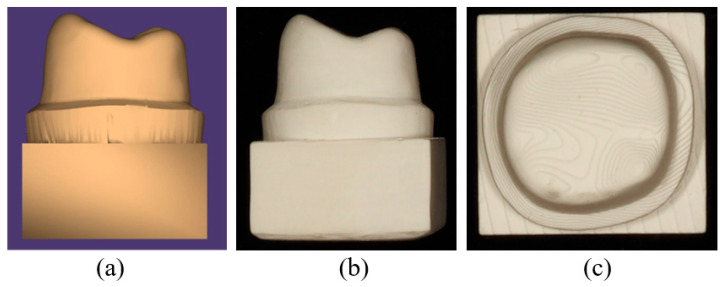
(**a**) STL file of resin die designed in Autodesk Fusion 360 CAD software; (**b**) distal view of the resin die; (**c**) occlusal view of the resin die.

A resin-die STL file of a mandibular first molar was designed in Autodesk Fusion 360, version 2.0.16786 (Autodesk Inc., San Francisco, CA, USA) according to standard full-crown preparation guidelines. Preparation parameters were described previously [27]. A corresponding molar crown with a 1 mm occlusal thickness was modeled in 3Shape Dental System (version 2020), with a 50 µm cement space between the crown intaglio and the die surface (Figure 2).

### 2.3. Fabrication of CRP Crown Specimens and Cementation

A total of 33 CE crowns were milled using the CARES M Series system (Straumann, Switzerland) with 1.0, 1.4, and 1.8 mm burs, which were replaced after every 17 crowns. For the additively manufactured groups, 33 VS and 33 HS crowns were fabricated using a DLP-based 3D printer (Freeform Pro 2, ASIGA, Ann Arbor, MI, USA). All crowns were designed as CAD models, exported as STL files, and processed using the manufacturer’s proprietary software. Printing was performed using the manufacturer’s recommended settings, including a 50 µm layer thickness, a 12.816 s exposure time, and a light intensity of 7.6 mW/cm^2^.

**Figure 3:**
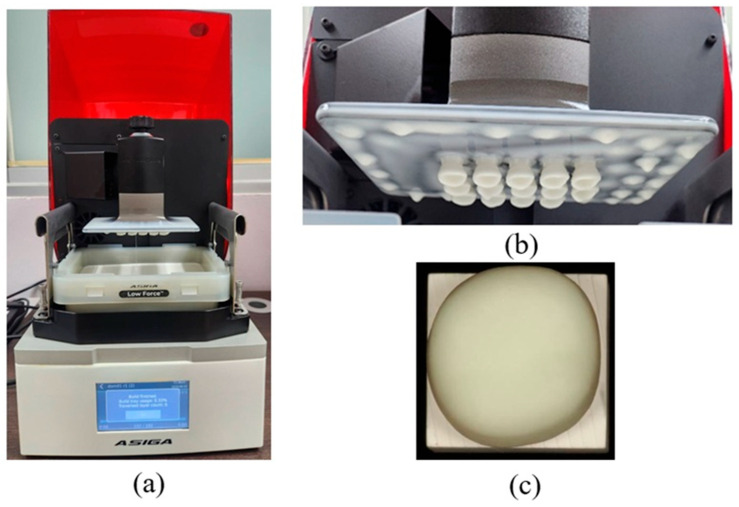
(**a**) The DLP-based Freeform Pro 3D printer (ASIGA; Anaheim Hills, USA). (**b**) The appearance of Hassawat-01 (HS) crowns after the printing process; (**c**) occlusal view of HS crown.

Following fabrication, the printed crowns underwent standardized post-processing. Support structures were removed using a cutting wheel or side cutters, and all surfaces were then airborne-particle abraded with 50 µm alumina powder at 1.5 bar for 10 s. All specimens were sequentially cleaned in 96% ethanol in an ultrasonic bath and air-dried. Post-curing was performed in a nitrogen atmosphere using a BEGO Otoflash device (BEGO, Bremen, Germany). equipped with two xenon stroboscopic lamps operating at 10 Hz and emitting light between 300 and 700 nm. Each printed crown underwent two post-curing cycles of 1500 flashes, followed by a 3 to 5 min cooling period until reaching room temperature [41]. To ensure methodological consistency, the experimental HS material was processed using the same equipment, post-curing protocol, and software workflow as the commercial VS material. This approach facilitates practical implementation by allowing HS to be fabricated under the same standard conditions used for clinically available DLP-printed CRP materials (Figure 3). All crowns, both milled and 3D-printed, were thermally conditioned in a heating cabinet (LEEC Ltd., Nottingham, UK) at 37 °C for 24 h before cementation.

All crown internal surfaces were sandblasted with 50 µm alumina powder as described above. Each material group was divided into three subgroups and luted with Maxcem Elite^®^, RelyX Unicem^®^, or Ketac Cem^®^, following the respective manufacturers’ instructions. Crowns were seated on resin dies under a standardized 20 N static load using a digital force gauge (SF-500; Wenzhou Sanhe Measuring Instrument Co., Ltd., Wenzhou, China) mounted on a manual screw stand to ensure uniform pressure [27,47,48]. This load level is consistent with previous crown-cementation studies and was selected to minimize variability in cement film thickness and internal gap geometry that can occur when seating forces differ between specimens. Excess cement was removed before polymerization. For Maxcem Elite^®^ and RelyX Unicem^®^, polymerization was performed using an LED curing unit (470 nm, 1000 mW/cm^2^; SmartLite Focus, Dentsply Sirona, Charlotte, NC, USA). Each specimen was light-cured for 40 s on all axial surfaces (mesial, distal, buccal, lingual) and then from the occlusal surface. For Ketac Cem^®^, crowns were left undisturbed for 3.5 min to allow self-setting. All specimens were stored at 37 °C for 24 h before micro-CT analysis.

### 2.4. Micro-Computed Tomography (Micro-CT) Analysis

Micro-CT imaging was performed to qualitatively evaluate internal cement morphology, with particular emphasis on voids or crack-like features in the occlusal region. After micro-CT acquisition, one representative specimen from the nine experimental groups was selected for descriptive comparison. All scans were obtained using a high-resolution micro-CT system (SkyScan 1172; Bruker, Belgium) with the following parameters: 67 kV, 41 μA, a 10 µm voxel size, and a 1 mm aluminum filter. Specimens were positioned perpendicular to the X-ray beam, and images were collected throughout a 360° rotation with a 0.2° rotation step. Projection images were reconstructed into cross-sectional slices using NRecon software (v1.6.9; Bruker) [27]. The reconstructed datasets were used exclusively to visualize cement distribution and internal structural morphology.

### 2.5. Fracture Resistance Test

**Figure 4:**
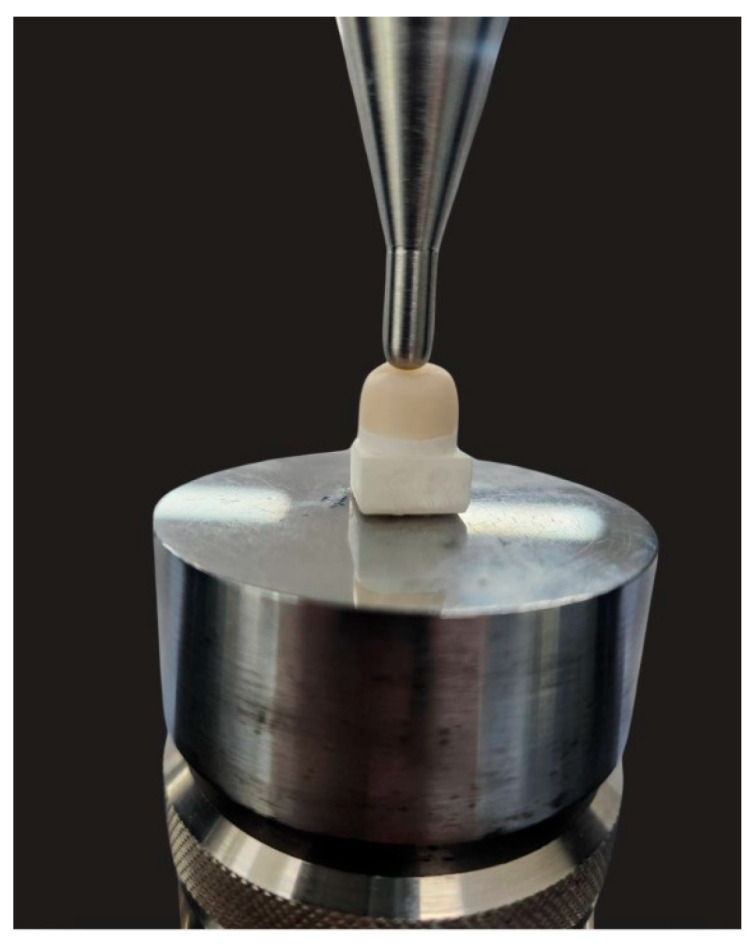
Position of the CRP crown specimen in the setup for single-load fracture test.

Fracture testing was performed using a universal testing machine (Shimadzu, Kyoto, Japan) with a 5 mm stainless steel ball positioned perpendicular to the central pit of each crown (Figure 4). A compressive load was applied at a crosshead speed of 1 mm/min until either a visible crack or a complete fracture occurred, and loading was terminated at a maximum of 2000 N [27]. For each specimen, the maximum fracture load and the corresponding failure mode were recorded. An intact specimen was defined as one with no visible crack or structural discontinuity. A crack was defined as a visible line or fissure within the material without complete separation, and a fracture was defined as catastrophic failure in which the crown separated into two or more distinct fragments.

### 2.6. Statistical Analysis

No failures occurred in CE, VS, or HS crowns luted with Maxcem Elite^®^ or RelyX Unicem^®^, all of which withstood loads exceeding 2000 N. These specimens were treated as right-censored and excluded from statistical analysis. In the Ketac Cem^®^ groups, four of eleven CE specimens exhibited fractures; however, all failure loads also exceeded 2000 N and were therefore also classified as right-censored. Both fracture and crack loads were recorded, and crack initiation was considered a valid failure value. Accordingly, only specimens with failure loads of 2000 N or less, whether cracked or fully fractured, were included in the statistical analysis. As a result, inferential comparisons were limited to VS and HS crowns luted with Ketac Cem^®^, which exhibited measurable failure within the load limit. An independent-samples *t*-test (α = 0.05) was used to compare their mean fracture resistance. The failure classification of each specimen (intact, cracked, or fractured) is summarized in Table 2.

## 3. Results

### 3.1. Fracture Resistance

**Figure 5:**
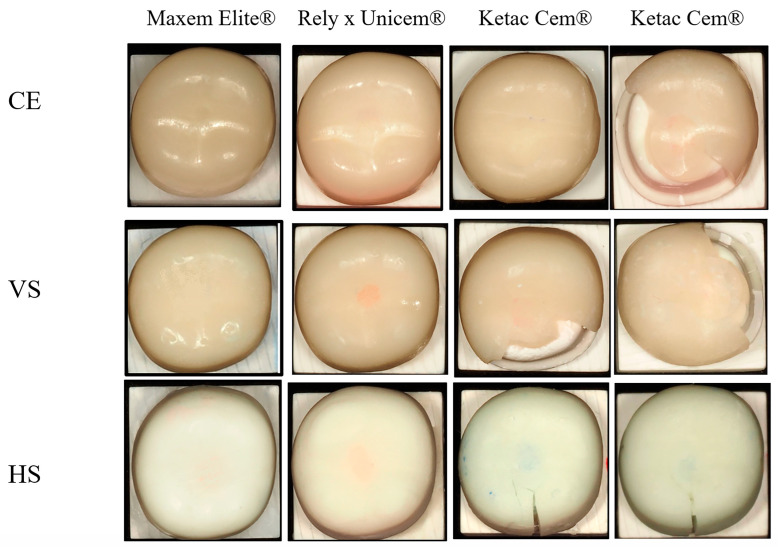
Post-fracture surface appearance of CRP crowns cemented with three luting agents. CE = Cerasmart^®^ 270; VS = VarseoSmile Crown Plus^®^; HS = Hassawat-01. Rows represent the crown materials ((**top**): CE; (**middle**): VS; (**bottom**): HS), and columns represent the cement types (Maxcem Elite^®^, RelyX Unicem^®^, and two representative Ketac Cem^®^ specimens). Each image illustrates the characteristic failure pattern for that material–cement combination. Resin-cemented groups (Maxcem Elite^®^ and RelyX Unicem^®^) generally showed minimal surface disruption, whereas Ketac Cem^®^ frequently produced visible crack lines, surface chipping, and cohesive cement remnants.

All crowns luted with Maxcem Elite^®^ and RelyX Unicem^®^ withstood the 2000 N load limit without visible cracks or fractures. In contrast, the Ketac groups exhibited lower fracture resistance and distinct failure modes. In the CE group, four of eleven crowns fractured slightly beyond 2000 N. Although mean values exceeded 2000 N, fractures occurred two to three seconds after the load limit was reached. VS-Ketac specimens showed seven fractures and four cracks (mean: 1795.2 ± 156.7 N), while all HS-Ketac specimens exhibited cracks without complete fracture (mean: 1732.6 ± 127.3 N) (Table 2). Representative failures are shown in Figure 5. The horizontal rows represent each ceramic material luted with different cements, whereas the vertical columns represent each ceramic material luted with the same cement. Due to the variation in fracture patterns among ceramics within the Ketac Cem^®^ group, this group is presented in two vertical rows to clearly illustrate the differences.

### 3.2. Micro-CT Analysis

**Figure 6:**
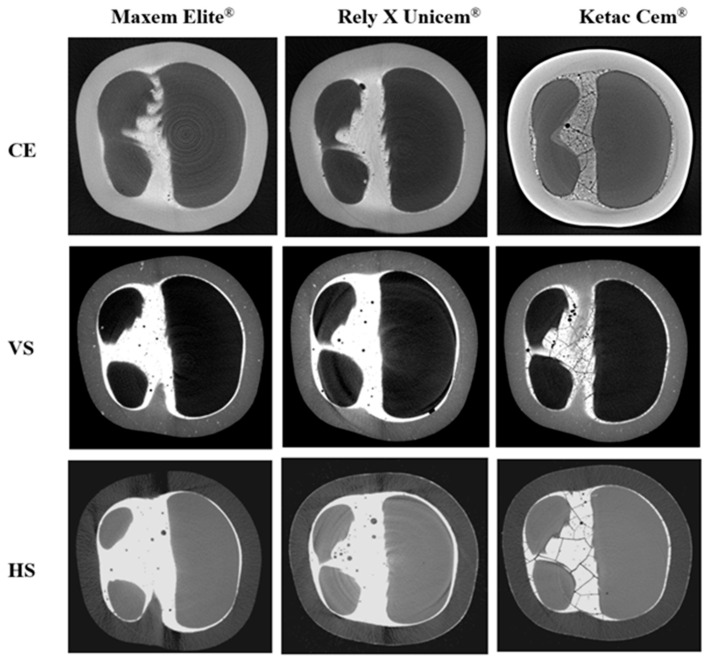
Micro-CT cross-sections showing internal cement morphology of CRP crowns after fracture testing. CE = Cerasmart^®^ 270; VS = VarseoSmile Crown Plus^®^; HS = Hassawat-01. Rows indicate crown materials (CE, VS, HS), and columns indicate luting cements (Maxcem Elite^®^, RelyX Unicem^®^, and Ketac Cem^®^). Resin cements (Maxcem Elite^®^ and RelyX Unicem^®^) produced uniform, low-void interfaces, while Ketac Cem^®^ showed extensive voids, crack-like defects, and irregular distribution, especially in DLP-printed VS and HS crowns.

Micro-CT images (Figure 6) revealed distinct differences in cement integrity. Maxcem Elite^®^ showed dense and homogeneous layers with minimal localized voids. RelyX Unicem^®^ displayed slightly more voids but retained a continuous and radiopaque interface. In contrast, Ketac Cem^®^ exhibited reduced radiopacity, uneven cement distribution, and numerous voids and crack-like defects, indicating poor adaptation and compromised structural integrity.

## 4. Discussion

This study investigated the influence of CRP material type and the elastic modulus of the luting cement on fracture resistance. It was hypothesized that differences in filler content, matrix composition, and modulus compatibility at the adhesive interface could affect performance under load. Both null hypotheses predicting no effect were rejected. All crowns luted with the resin-based cements Maxcem Elite^®^ and RelyX Unicem^®^ withstood loads greater than 2000 N without failure, whereas those luted with Ketac Cem^®^ showed lower fracture resistance and visible failures, especially in the DLP-printed groups. These findings confirm that both the material type and the cement significantly influence fracture resistance.

Filler composition played a decisive role in the fracture behavior of the CRP crowns. CE, a milled hybrid ceramic, contains 71 wt.% nanofillers, consisting of 20 nm silica and 300 nm barium-glass (BG) particles [49]. Because BG has a refractive index compatible with the resin matrix (RI approx. 1.53), it can enhance optical homogeneity, radiopacity, and mechanical strength [50,51,52,53]. These properties likely contributed to the superior fracture resistance observed in CE-Ketac specimens. In contrast, VS is a DLP-printed composite containing 30 to 50 wt.% silanized glass fillers (approx. 0.7 µm) embedded in a UDMA/TEGDMA/Bis-EMA matrix and polymerized with diphenyl 2,4,6-trimethylbenzoyl phosphine oxide (TPO) initiators [50,54,55]. Although the absence of BG in VS may contribute to its lower fracture resistance, this interpretation should be viewed cautiously. CE and VS differ in both filler composition and filler loading, making it difficult to isolate the effect of any single variable. In the current HS prototype, filler composition also remains a confounding factor. The individual effects of BG and alumina on mechanical behavior cannot yet be distinguished. To clarify their roles, HS will need to be reformulated in several controlled versions, for example, with and without BG and with varying amounts of alumina, while keeping viscosity within the acceptable DLP printing range. Such targeted formulation studies are essential for identifying the contribution of each filler and should be a primary focus of future work.

HS, another DLP-printed material, comprises 38 wt.% non-silanized glass and alumina fillers with an average particle size of 0.7 µm. Although alumina reinforcement is associated with favorable wear resistance and biocompatibility [5,56], its inherently high hardness, low fracture toughness, and lack of silane coupling likely contributed to the superficial crack patterns, rather than catastrophic fractures, observed in the HS–Ketac specimens. The lower filler loading and higher resin fraction relative to CE may also explain the reduced fracture resistance of HS. Consequently, HS exhibited lower mechanical strength than CE but did not differ significantly from VS, while its fracture loads remained above the maximum physiological masticatory forces reported for natural dentition. It should be noted that although higher filler content generally enhances mechanical properties by reinforcing the resin matrix, it also increases viscosity and promotes particle agglomeration, which can impair material homogeneity and compromise mechanical reliability. HS demonstrated a printable viscosity of approximately 1.054 Pa·s, measured using a rotational Brookfield DV-E viscometer (Brookfield Viscometers Ltd., Harlow, UK; controlled RPM, room temperature conditions), which falls within the accepted viscosity window for DLP printing (0.4 to 3.5 Pa·s) [57,58,59].

In addition to filler characteristics, the polymer matrix critically influences the mechanical performance of CRP materials. CE features a cross-linked network of UDMA, Bis-MEPP, and dimethacrylates, producing a rigid yet moderately flexible matrix suitable for milled restorations [60]. This likely accounts for the delayed fractures in CE specimens, occurring seconds after peak load and suggesting gradual crack propagation and internal stress redistribution. VS, optimized for DLP printing, uses Bis-EMA, which allows low viscosity and high light transmission. Combined with TPO-based initiators, this formulation enables rapid and high-resolution curing [61,62,63]. However, the resulting resin matrix may exhibit lower cross-link density and reduced toughness, contributing to the catastrophic fractures seen in VS-Ketac specimens. HS incorporates a resin matrix dominated by Urethane acrylate (UA) and supplemented by 2-phenoxyethyl acrylate, tripropylene glycol diacrylate, and trimethylolpropane triacrylate (61.5 wt.% total resin) to improve elasticity and fracture toughness [19]. This accounts for the plastic deformation and surface cracking, rather than complete failure, observed in HS crowns. However, UA systems have reduced rigidity, limited light penetration, and poorer esthetics, particularly when combined with opaque fillers such as alumina [5,56]. These findings highlight the role of the matrix in stress absorption and redistribution. CE benefits from its stiff cross-linked network. VS prioritizes printability at the expense of strength, whereas HS achieves crack resistance through greater ductility but sacrifices rigidity. To optimize HS, matrix–filler interactions could be enhanced through zirconia co-reinforcement [64,65,66,67] or the incorporation of barium-glass (BG) fillers to boost strength, radiopacity, and esthetics [50,51,52,53]. Raising BG or bioactive filler content to 60 wt.% or more may align these materials with ADA ceramic criteria and improve overall performance [57,68,69].

Beyond ceramic composition, our findings confirm that the elastic modulus of the luting cement is a critical determinant of fracture resistance in CRP crowns, consistent with prior FEA studies showing that cement stiffness strongly influences intra-ceramic stress distribution [31,32,33,38]. In this study, crowns luted with resin cements of intermediate-to-high modulus, Maxcem Elite^®^ approx. 4 GPa and RelyX Unicem^®^ approx. 13 GPa) consistently withstood loads exceeding 2000 N. In contrast, the stiffer glass-ionomer (GI) cement (Ketac Cem^®^ approx. 20 GPa), markedly reduced fracture resistance. The poor performance of Ketac Cem^®^ can be attributed not only to its micromorphological behavior but also to its chemical bonding mechanism. Glass-ionomer cements rely on an acid–base reaction between polyacrylic acid and fluoroaluminosilicate glass to form ionic bonds. Although this enables limited adhesion to tooth substrates, Ketac does not bond chemically to silanized ceramic or resin-based CRP surfaces. In contrast, resin cements form covalent bonds with both ceramics and primed dentin, creating a stronger and reinforced monobloc that improves load transfer and fracture resistance [70]. In addition to weaker bonding, the relatively high elastic modulus of Ketac Cem^®^ concentrates tensile stresses at the crown–cement interface rather than dissipating them through the restoration. This combination of insufficient adhesion and unfavorable stress distribution explains the significantly lower fracture loads observed in Ketac-luted specimens, especially in the DLP-printed CRP groups. These findings align with previous studies on lithium disilicate and zirconia-reinforced ceramics, which also reported the lowest failure loads when glass-ionomer cements were used and recommended against their use for bonded ceramic restorations [70,71]. Our results differ from those of Al-Wahadni et al., who found minimal influence of cement type when testing IPS Empress and In-Ceram crowns on natural premolars [72]. This discrepancy likely reflects the heterogeneity of natural dentin, whose variable modulus, hydration, and tubule structure can mask cement-related mechanical effects. In the present study, standardized resin dies were intentionally used to minimize substrate variability, allowing the influence of cement stiffness and bonding mechanisms to be observed more clearly. Nevertheless, validation of using natural teeth is still needed to confirm clinical relevance under more complex biomechanical conditions.

Overall, the findings indicate that resin cements with an intermediate-to-high elastic modulus improve the fracture resistance of CRP-based crowns more effectively than glass-ionomer cement. This supports the recommendations of Lia et al. [38] and Shahrbaf et al. [37], who proposed that intermediate-modulus cements reduce interfacial stress and prevent excessive deformation, whereas very low modulus cements (approx. 1.8 GPa) provide insufficient mechanical support. Importantly, our results challenge the assumption that a higher cement modulus universally improves fracture behavior. Although Dong et al. [29] reported reduced ceramic stress when stiff cements were modulus-matched to ceramic, the high-modulus Ketac Cem^®^ increased internal stress within the cement layer and promoted cohesive failure in the present study. This outcome is consistent with Davidson et al. [73], who reported microcracking and structural disruption in thin GI layers due to shrinkage. While Ha et al. [39] reported acceptable performance of GI under zirconia crowns, the lower-modulus CRP materials used in our study (CE approx. 9.6 GPa; VS approx. 4.4 GPa; and HS approx. 4.75 GPa) performed more favorably when luted with resin cements of compatible modulus. Collectively, these results reinforce the view highlighted by Thompson et al. [34] that interfacial stiffness and adaptation are more important than reliance on highly rigid cements such as zinc phosphate [29,34,73]. Resin cements with an intermediate-to-high elastic modulus appear to provide the most favorable balance, provided that viscosity and cement thickness are properly controlled.

Micro-CT analysis provided visual confirmation that supported the observed mechanical behavior. Crowns luted with Maxcem showed the fewest internal voids, followed by those luted with RelyX, whereas Ketac displayed the highest void volume and numerous crack-like defects. These flaws likely served as stress concentrators, contributing to micromotion and weakening of the interface [74,75,76,77]. Although RelyX showed slightly more voids than Maxcem, the absence of radiographic cracks suggests that these voids were likely entrapped microbubbles rather than stress-induced failures [78,79]. In contrast, the extensive voids and cracks observed in Ketac may be attributed to its high stiffness, limited flowability, and poor chemical adhesion to low-modulus CRPs. Under thin-film conditions, GI cements are known to undergo shrinkage-induced microcracking, particularly when bonded to compliant substrates such as polymer-based ceramics [73]. Clinically, despite offering chemical bonding potential, GI cements lack sufficient fracture toughness and are susceptible to internal fracturing under functional occlusal loads [39,73]. It is also important to note that the potential chemical bonding of GI cements, typically observed when interacting with dentin, may have been limited in this study due to the use of resin dies, which do not provide ionic bonding sites like natural tooth substrates. This material mismatch may have further contributed to the weak interfacial adaptation and reduced stress distribution observed in the GI groups. Resin-based cements, by contrast, demonstrated better interfacial adaptation and internal homogeneity, reinforcing their role in enhancing the fracture resistance of CRP restorations.

Although the micro-CT assessment was qualitative, it effectively illustrated morphological differences between the cement groups that correlated with mechanical outcomes. The scanning parameters used in this study (67 kV, 41 µA, 10 µm voxel size) offer sufficient resolution for more detailed morphometric analysis. Future studies could incorporate CTAn-based three-dimensional volumetric analysis to quantify porosity-related parameters, such as void volume, void fraction, and void number density, within the cement interface. This quantitative approach will enable a more comprehensive understanding of how internal microstructure influences fracture behavior across different crown–cement systems.

In this study, VS crowns cemented with RelyX Unicem^®^ withstood loads greater than 2000 N, markedly higher than the 1629 ± 118 N reported by Suksuphan et al. [27] for the same material-cement combination. This discrepancy may be attributable to differences in pre-testing protocols. In our study, specimens were stored dry at 37 °C before micro-CT and mechanical testing, whereas Suksuphan et al. stored their specimens in water. Water exposure can compromise the mechanical integrity of CRP materials and luting cements, particularly moisture-sensitive GI types, by accelerating cement dissolution, reducing cohesion, and weakening the filler–matrix bond [80,81,82]. Previous studies show that water storage and thermocycling induce microcrack formation and structural degradation in CRPs, primarily through polymer plasticization, swelling, and silane hydrolysis [81,82,83,84]. Materials with higher resin content are especially vulnerable, due to increased water uptake promoting microcrack propagation, filler debonding, and esthetic deterioration [85]. Thus, the absence of water immersion in our study likely preserved the structural integrity of RelyX Unicem^®^, which may account for the higher fracture resistance observed. These findings underscore the importance of environmental conditions and specimen handling, in addition to material and cement selection, when evaluating CRP–cement performance.

These findings highlight that both the elastic modulus of the luting cement and the ceramic composition of CRP materials critically influence fracture resistance. Within the limitations of this study, all CRP crowns luted with resin-based cements withstood forces exceeding 2000 N. HS, a newly developed CRP composite, showed high crack resistance despite its esthetic limitations, supporting its potential use as a long-term interim crown. The high fracture resistance observed may be attributed to its polymer-rich matrix, which enhances energy absorption and crack deflection. However, this in vitro study was conducted under idealized laboratory conditions that excluded water storage, thermocycling, and complex occlusal loading, which limits direct clinical extrapolation.

As a first-generation prototype, HS still lacks comprehensive characterization data essential for material validation, including degree of conversion, filler–matrix interfacial bonding, polymer network quality, and long-term stability. This study focused on evaluating the fracture resistance of HS relative to two commercially available CRP crown materials as an initial step toward determining the viability of alumina-reinforced CRPs. Although alumina was incorporated to enhance mechanical and optical properties, further investigation is needed to clarify its role in improving long-term strength, dimensional stability, and interfacial integrity, particularly under aging conditions that better simulate clinical use. Continued material optimization is necessary to achieve properties comparable to those of established CRP systems. Future studies should also assess the biomechanical behavior, optical performance (e.g., translucency, gloss, and color stability), and biocompatibility of HS. Potential strategies include alumina–zirconia co-reinforcement, incorporation of bioactive or radiopaque fillers, enhancement of filler–matrix bonding, and improvements in esthetic properties. In addition, testing on natural teeth is recommended, because resin dies do not replicate the biomechanical behavior of dentin. Complementary approaches such as finite element analysis (FEA), long-term fatigue testing, and clinical trials are also warranted to more comprehensively evaluate functional performance and clinical applicability.

## 5. Conclusions

Within the limitations of this study, fracture strength depended on both the ceramic type and the modulus of the luting cement. High-modulus cements were unsuitable for low-modulus CRP crowns due to increased interfacial stress and promoted void formation. CE and VS showed adequate strength for definitive restorations, whereas HS, with its alumina fillers and UA matrix, appeared more suitable for long-term provisional use. Although alumina reinforcement enhanced strength, it reduced the translucency, color stability, and surface gloss of HS.

## Figures and Tables

**Table 1:** Details of the materials used in this research [27,33,42,43,44,45,46].

Group	Product	Manufacturer	Composition	Elastic Modulus (Gpa)	Lot. No.
Milling	Cerasmart^®^ 270 (CE)	GC, Tokyo, Japan	Bis-MEPP, UDMA, dimethacrylate, silica (20 nm), and barium glass (300 nm) 71 wt.%	9.6	2108056
3D-Printing	VarseoSmile Crown Plus^®^ (VE)	Bego, Bremen, Germany	Esterification products of 4.4′-isopropylidiphenol, ethoxylated and 2-methylprop-2enoic acid. Silanized dental glass, methyl benzoylformate, diphenyl (2,4,6-trimethylbenzoyl) phosphine oxide. Total content of inorganic fillers (particle size 0.7 μm) is 30–50 wt.%	4.4	601817
	Hassawat-01 (HS)	Faculty of Science, Chulalongkorn University, Bangkok, Thailand	Urethane acrylate, 2-Phenoxyethyl acrylate, Tripropyleneglycol diacrylate, Trimethylolpropane triacrylate, Trimethyl benzoyl diphenyl phosphine oxide (61.5 wt.% total resin), and Aluminum oxide 0.7 μm 38.5 wt.%	4.75	N/A
Luting cement	Maxcem Elite^®^	SDS Kerr, Orange, CA, USA	Glyceroldimethacrylate dihydrogen phosphate (GPDM) hydroxyethylmethacrylat (HEMA), 4-methoxyphenol (MEHQ), cumolhydroperoxid (CHPO), methacrylate ester monomers, titanium dioxide, pigments	4	A159983
	RelyX Unicem^®^	3M ESPE, Saint Paul, MN, USA	Powder: glass fillers, silica, calcium hydroxide, self-curing initiators, pigments, light-curing initiators, substituted pyrimidine, peroxy compound. Liquid: methacrylated phosphoric esters, dimethacrylates, acetate, stabilizers, self-curing initiators, light-curing initiators	13.0	10684647
	Ketac Cem^®^	3M ESPE, Saint Paul, MN, USA	non-reactive zirconia silica filler, the methacrylated polycarboxylic acid, HEMA, BisGMA, water and potassium persulfate.	20	10605282
Silane Coupling Agent	RelyX Ceramic Primer^®^	3M ESPE, Saint Paul, MN, USA	Ethyl alcohol, water, 3-methacryloxypropyltrimethoxysilane	-	NF44358
Resin die	Rigid 10K Resin	Formlabs Inc., Somerville, MA, USA	Highly glass-filled resin-based methacrylate	14.9	N/A

N/A = not appliable.

**Table 2:** The number of fractured and cracked CRP crowns and means of fracture load.

Types of Ceramic	Luting Cement	Cracked Crowns (n)	Fractured Crowns (n)	Mean ± SD (N)	Min. (N)	Max. (N)
Cerasmart^®^ 270 (CE)	Maxem Elite^®^ (n = 11)	0	0	>2000	N/A	N/A
	Rely X Unicem^®^ (n = 11)	0	0	>2000	N/A	N/A
	Ketac Cem^®^ (n = 11)	0	4	2000	2000	2000
VarseoSmile Crown Plus^®^ (VS)	Maxem Elite (n = 11)	0	0	>2000	N/A	N/A
	RelyX Unicem^®^ (n = 11)	0	0	>2000	N/A	N/A
	Ketac Cem^®^ (n = 11)	4	7	1795.2 ± 156.7	1689.9	1980.0
Hassawat-01 (HS)	Maxem Elite^®^ (n = 11)	0	0	>2000	N/A	N/A
	RelyX Unicem^®^ (n = 11)	0	0	>2000	N/A	N/A
	Ketac Cem^®^ (n = 11)	11	0	1732.6 ± 127.3	1540.6	1875.6

(N) = newton; (n) = number of specimens; SD = standard deviation; N/A = not applicable.

## Data Availability

The original contributions presented in this study are included in the article. Further inquiries can be directed to the corresponding author.

## Abbreviations

The following abbreviations are used in this manuscript:

CRP	Ceramic-Reinforced Polymer
CE	Cerasmart^®^ 270
VS	VarseoSmile Crown Plus^®^
HS	Hassawat-01
BG	Barium-glass
UA	Urethane acrylate
GI	Glass ionomer cement
UDMA	Urethane dimethacrylate
